# Characterization of the complete chloroplast genome of *Linnaea borealis*, a rare, clonal self-incompatible plant

**DOI:** 10.1080/23802359.2019.1698995

**Published:** 2019-12-12

**Authors:** Hairui Liu, Mingze Xia, Qingmeng Xiao, Jie Fang, Anqi Wang, Shilong Chen, Dejun Zhang

**Affiliations:** a State Key Laboratory of Plateau Ecology and Agriculture, Qinghai University, Xining, China;; b College of Eco-Environmental Engineering, Qinghai University, Xining, China;; c Key Laboratory of Adaptation and Evolution of Plateau Biota, Northwest Institute of Plateau Biology, Chinese Academy of Sciences, Xining, China

**Keywords:** *Linnaea borealis*, complete chloroplast genome, phylogenetic analysis

## Abstract

*Linnaea borealis* L. is a creeping shrub which grows about 5–10 cm high and is a rare clonal plant. *Linnaea* is a monotypic genus. Here, we release and detail the complete chloroplast genome sequences of *L. borealis*, whose size is 161,576 bp, containing a large single copy region (LSC) of 85,609 bp and a single copy region (SSC) of 46,694 bp which typically separates by a pair of inverted repeats (IRs) of 29,210 bp. The amount of the overall genes is 136, which includes 37 tRNA genes, eight rRNA genes, and 91 protein-coding genes. The content of the G/C in whole plastome is 61.74% while the G/C content of the LSC, SSC, and IR region are 36.58%, 38.86%, and 42.25%, respectively. The complete cp genome sequences of *L. borealis* will be a useful resource to the phylogenetics study in family Caprifoliaceae.

*Linnaea borealis* L. is a creeping and evergreen shrub belongs to family Caprifoliaceae, usually 5–10 cm tall, generally distributing in the areas with high altitude and cold climate in northern China such as Hebei, Heilongjiang, Jilin, Liaoning, Nei Mongol, Xinjiang (Xu et al. 1988). It is a very precious species, known as rare clonal plant (Hou et al. 2013; Wiberg et al. 2016; Zhai et al. 2016). It is undergoing a considerable decline in China, Scotland, and some other European countries (Scobie and Wilcock 2009). It is the only species in the genus *Linnaea*. According to the previous studies, the evolution of *L. borealis* reaches a high level in the family Caprifoliaceae, but the conclusion has not been proved from the complete chloroplast genome before (Zhu et al. 2007; Zhou 2012). Our study releases the complete cp genome sequence of the *L. borealis*, and it would not only promote the phylogenetics study in family Caprifoliaceae but also provide useful genetic information for the protection of *L. borealis*.

The samples we studied were collected in the Changbaishan mountain (128°10′31.05″E, 42°10′56.28″N) and the voucher specimens (liu2019045) are deposited in the Herbarium of Northwest Institute of Plateau Biology (HNWP), Chinese Academy of Sciences, Xining, China. The extraction and assembly were finished by the Genepioneer Biotechnologies (Nanjing, China). To annotate the cp genome sequence, the Geseq (https://chlorobox.mpimp-golm.mpg.de/geseq.html) has been utilized (Tillich et al. 2017). With the help of Sequin v.15.10, the correction was finished manually (Liu et al. 2012). The complete chloroplast genome sequence of the *L. borealis* we released has already been accepted by the GenBank and the accession number is MN548092.

The length of the complete cp genome of the *L. borealis* we reported is 161,576 bp and the whole genome contains 136 genes, which includes 37 tRNA genes, eight rRNA genes, and 91 protein-coding genes. The genome was a quadripartite circular DNA containing two inverted repeats (IRs) of 29,210 bp, a large single copy region (LSC) of 85,609 bp and a single copy region (SSC) of 46,694 bp. The content of the G/C in whole plastome is 61.74%, comparing to the G/C content of the LSC, SSC, and IR region are respectively 36.58%, 38.86%, and 42.25%. In IR, 10 unique protein-coding genes (*rps*3, *rpl*22, *rps*19, *rpl*2, *rpl*23, *ycf*2, *ndh*B, *rps*7, *ycf*15, and *ycf*1), seven unique tRNA(*trn*I-CAU, *trn*L-CAA, *trn*V-GAC, *trn*I-GAV, *trn*A-UGC, *trn*R-ACG, and *trn*N-GUU), and four unique rRNA (rrn16s, rrn23s, rrn4.5s, and rrn5s) have been coded.

Twenty-two published complete chloroplast genome sequences have been used to the analysis of the phylogenetic position relationship between *L. borealis* and other species of Caprifoliaceae. All the complete cp genome sequences we used to construct the maximum-likelihood tree were aligned using the MAFFT v7.017 (Kazutaka and Standley 2013). We use the Jmodeltest and chose the GTR + R + I model as the reference to make the construction of the ML tree in raxmlGUI 1.5b1 (Diego et al. 2012; Silvestro and Michalak 2012). According to the result, the *L. borealis* has close relationship with the genus *Kolkwitzia* and *Dipelta* (Figure 1).

**Figure 1.**
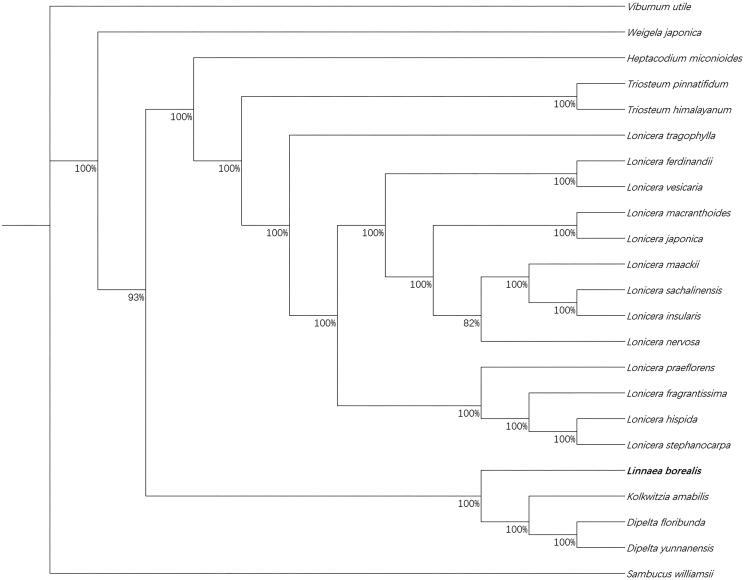
The phylogenetic tree of Caprifoliaceae based on *Linnaea borealis* and other related 22 species in the Caprifoliaceae established by maximum-likelihood method. The accession number of the chloroplast genome sequences the study used: *Dipelta floribunda*, NC_037955.1; *Dipelta yunnanensis*, NC_042201.1; *Heptacodium miconioides*, NC_042739.1; *Kolkwitzia amabilis*,NC_029874.1; *Lonicera ferdinandii*,NC_040963.1;*Lonicera fragrantissima*, MG738669.1; *Lonicera hispida*, NC_040962.1; *Lonicera insularis*, NC_039634.1; *Lonicera japonica*,MH028738.1; *Lonicera maackii*,NC_039636.1; *Lonicera macranthoides*,NC_040959.1; *Triosteum himalayanum*, MN_551173; *Lonicera nervosa*, NC_040961.1; *Lonicera praeflorens*, NC_039635.1; *Lonicera sachalinensis*, NC_039637.1; *Lonicera stephanocarpa*, NC_037954.1; *Lonicera tragophylla*, NC_037953.1; *Lonicera vesicaria*, MH_028743.1; *Sambucus williamsii*, NC_033878.1; *Triosteum pinnatifidum*, NC_037952.1; *Viburnum utile*, NC_032296.1; *Weigela japonica,* MK_397907.1.
